# Molecular and Cellular Networks in The Suprachiasmatic Nuclei

**DOI:** 10.3390/ijms20082052

**Published:** 2019-04-25

**Authors:** Lama El Cheikh Hussein, Patrice Mollard, Xavier Bonnefont

**Affiliations:** Institut de Génomique Fonctionnelle (IGF), University Montpellier, CNRS, INSERM, 34094 Montpellier, France; Lama.El-Cheikh@igf.cnrs.fr (L.E.C.H.); Patrice.Mollard@igf.cnrs.fr (P.M.)

**Keywords:** circadian rhythms, neuronal networks, in vivo imaging, jetlag

## Abstract

Why do we experience the ailments of jetlag when we travel across time zones? Why is working night-shifts so detrimental to our health? In other words, why can’t we readily choose and stick to non-24 h rhythms? Actually, our daily behavior and physiology do not simply result from the passive reaction of our organism to the external cycle of days and nights. Instead, an internal clock drives the variations in our bodily functions with a period close to 24 h, which is supposed to enhance fitness to regular and predictable changes of our natural environment. This so-called circadian clock relies on a molecular mechanism that generates rhythmicity in virtually all of our cells. However, the robustness of the circadian clock and its resilience to phase shifts emerge from the interaction between cell-autonomous oscillators within the suprachiasmatic nuclei (SCN) of the hypothalamus. Thus, managing jetlag and other circadian disorders will undoubtedly require extensive knowledge of the functional organization of SCN cell networks. Here, we review the molecular and cellular principles of circadian timekeeping, and their integration in the multi-cellular complexity of the SCN. We propose that new, in vivo imaging techniques now enable to address these questions directly in freely moving animals.

## 1. Introduction

Circadian clocks are exquisitely adapted to the natural cycle of days and nights. As the result of 2.5 billion years of evolution [1], they afford an adaptive advantage to living organisms in response to the daily variations of their natural environment [2,3]. Ironically, as human beings, our internal clock has turned into a disadvantage in our modern 24/7 western society. Despite the invention of artificial light and jet planes that bridge the opposite sides of the planet within a few hours, we are not readily able to break free from the natural 24 h day, imposed by the rotation of our planet since the origin. As a consequence, people travelling across time zones suffer from jetlag, and night-shift workers are threatened by critical health problems, such as mood disorders, obesity, and metabolic syndrome, increased risk of heart failure, and cancer [4,5,6,7,8]. Thus, one may now wonder whether loosening our clock would enable us to better cope with our societal constraints [9].

In mammals, the central circadian pacemaker is located in the suprachiasmatic nuclei (SCN) of the hypothalamus [10,11], in which its precision and robustness emerge from the circuit-level organization of cell-autonomous circadian oscillators [12,13,14,15]. As such, understanding the multi-cellular makeup of the SCN constitutes an imperative challenge on the way to fight jetlag and circadian disorders. Here, we review the basic principles that govern circadian rhythmicity at the cellular level, how clock cells form a complex assembly within the SCN, and their functional interactions. Finally, we propose further perspectives into how new techniques in imaging will enable to address the circuit-level organization of the SCN in vivo.

## 2. General Principles of Cell-Autonomous Circadian Timekeeping

The long quest to identify genetic determinants and to decipher the molecular mechanisms of circadian timekeeping, as recognized by the Nobel Committee for Physiology or Medicine, has been reviewed extensively elsewhere [16,17]. It is very remarkable that the whole clock machinery ticks at the single-cell level, as it mostly relies on intracellular mechanisms, such as the complex interplay between positive and negative regulators within transcription–translation feedback loops (TTFL). In mammals, the positive and negative arms of the molecular core clock are the transcription factors circadian locomotor output cycles protein kaput (CLOCK) and brain and muscle ARNT-like1 (BMAL1), and the proteins period (PER) and cryptochrome (CRY), respectively. The CLOCK–BMAL1 heterodimer binds to enhancer box (E-box) regulatory sequences to promote the expression of PER and CRY, which assemble into protein complexes. The PER–CRY complexes are hyper-phosphorylated by casein kinases, and translocate into the cell nucleus where they accumulate and finally repress their own expression. The degradation of PER–CRY complexes and the repression of their production eventually enables the whole process to reinitiate, nearly 24 h after the first cycle started. An accessory interlocked loop, involving nuclear receptors of the REV–ERB family, stabilizes the cycle. Although non-TTFL mechanisms have also been described [18,19,20], these molecular loops compose the major self-sustained circadian drive in mammalian nucleated cells, even when they are isolated from the rest of the organism [21,22].

This molecular clock drives circadian rhythms in gene expression, cell metabolism, and physiology, in virtually all our cells. In addition to their essential participation within the clockwork itself, the transcription factors CLOCK–BMAL1 and REV–ERBs pace the circadian transcription of many target genes through binding to their respective recognition sequences. These so-called first-order clock-controlled genes (CCGs) encode other transcription factors that, in turn, control the circadian expression of second- and third-order CCGs [16,23]. Beyond this transcriptional cascade, it has become evident that post-transcriptional mechanisms also control the circadian abundance of mRNA in the cell nucleus [24,25]. Moreover, different organs exhibit remarkably different circadian transcriptomes, as promoted by tissue-specific interactions of BMAL1 with non-circadian transcription factors [26]. Thus, while 5–15% of the mRNA expressed in a given organ follow a circadian rhythm of accumulation [27,28], more than 80% of the protein-coding genes appear to be expressed in a circadian manner somewhere in the body [29]. This suggests that most, if not all, of our genes are potential circadian clock targets, directly or indirectly.

More importantly, changes in the environment are able to alter the circadian clockwork in mammalian cells. Besides the E-box sequences involved in the circadian TTFL, Period genes also contain other regulatory sequences in their promoter that modulate their expression through several signaling pathways. For example, an increase in cytosolic calcium or cAMP (cyclic adenosine monophosphate)concentration [30,31], the activation of the nuclear glucocorticoid receptor [32,33,34,35], or a physiological increase in temperature [36,37,38] mediate the fast induction of the Per genes through conserved cyclic AMP response elements (CRE), glucocorticoid response elements (GRE), or heat shock elements (HSE), respectively. Moreover, temperature cycles also regulate the amplitude of circadian gene expression by post-transcriptional mechanisms [39]. Therefore, a wide array of environmental cues can modulate and rapidly reset the circadian cell rhythmicity.

## 3. Cell-Autonomous Circadian Timekeeping in SCN Neurons

Unsurprisingly, individual SCN neurons express the circadian molecular clockwork, as most of our cells do. As in other organs, circadian gene expression in the SCN encompasses various cellular functions, from cell metabolism to protein biosynthesis and degradation [29,40]. Interestingly, several secreted factors, such as arginine vasopressin (AVP) [41], vasoactive intestinal polypeptide (VIP) [42], or prokineticin 2 [43], and molecular players involved in vesicular secretion are under circadian regulation in the SCN, either at the transcriptomic or proteomic level [40,44]. Hence, both the secreting machinery and the vesicular cargo supposed to convey circadian timing to other brain regions are under clock control in SCN neurons, which supports the key role of the SCN as the central circadian pacemaker that coordinates all of the body rhythms [45].

Furthermore, SCN cell metabolism and physiology also exhibit circadian modulation. Individual SCN neurons display spontaneous circadian rhythms in membrane excitability, which underlie the circadian variations in action potential discharge rates [46]. Classically, the depolarized and hyperpolarized membrane states correspond to periods of high- and low-firing rates [47,48], respectively, but a number of SCN neurons sustain an uncommon depolarized state, responsible for the daily silencing of their spike activity [49]. The mechanisms and identity of the ionic conductances responsible for these circadian rhythms in neuronal activity have been extensively reviewed previously [48,50]. The circadian modulation of electrophysiological activity is cell-autonomous and depends on the integrity of the molecular clockwork, as shown by the altered rhythmicity of SCN neurons with genetically impaired or down-regulated clock gene expression [47,51,52,53]. An elegant bicycle model has been proposed, which accounts for the circadian variation in the membrane potential of SCN neurons. This mechanism, conserved between flies and mice, involves the antiphase regulation of a depolarizing sodium current and a hyperpolarizing potassium conductance [54]. Hence, the molecular clockwork coordinates rhythmic cell functions and processes at many different levels to ensure overt circadian outputs from master clock SCN neurons.

However, despite the unicity in the basic principles underlying circadian rhythmicity, single-cell activity patterns generated from SCN neurons are remarkably variable. Depending on culture conditions, 20–80% of individual SCN neurons exhibit circadian variations in electrophysiological firing [12,13,46,55], and some neurons can switch back and forth between the rhythmic and arrhythmic states [55]. Among rhythmic neurons, the measured circadian periods display great diversity, with differences of more than 2.5 h in cell pairs, even in the same culture [12,13,52]. Furthermore, the circadian period appears highly versatile from one cycle to the other in a same neuron [12]. This variability in single-neuron circadian rhythms indicates that the cell-autonomous rhythmicity in the SCN is rather unstable and unreliable. Thus, other mechanisms should be at work to confer robustness and precision to the central circadian pacemaker. Deciphering such mechanisms, beyond the molecular makeup of circadian cell oscillators, has become a new challenge, in order to understand how we could reconcile our clock with our modern society rhythms.

## 4. The Origin of Circadian Precision and Robustness within The SCN

Specifically, the properties that turn a community of individual cell oscillators into a coherent and reliable circadian pacemaker originate from the SCN itself [56,57]. The comparison of individual SCN neuron electrical rhythmicity in dispersed cell cultures and within SCN slices revealed that circadian precision increases significantly when the native tissue context is preserved. Both the cycle-to-cycle variability in single neurons, and the large differences in circadian periods between neurons appeared reduced in slices, giving rise to homogenous circadian firing of action potentials at the tissue level [12,13]. It is worth noting that the pharmacological silencing of SCN neuronal firing with the voltage-dependent sodium channel blocker, tetrodotoxin [15], or the chronic inhibition of exocytosis by Botox A [44], both profoundly disorganized the cellular circadian rhythmicity in SCN slices. Hence, intercellular coupling through the action potential-dependent release of signaling molecules contained in exocytosis vesicles contributes to the precision of individual circadian cell oscillators and to their coherence in the SCN.

**Figure 1:**
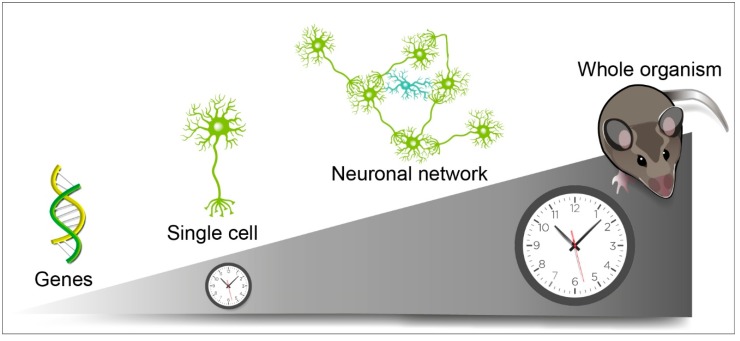
The robustness of circadian rhythms observed at the organism level emerges from cell networks in the suprachiasmatic nuclei (SCN). A genetically encoded clock drives self-sustained circadian rhythmicity in individual cells. Intercellular coupling in the SCN coordinates cell-autonomous rhythms into a coherent and resilient circadian pacemaker that tunes all the body rhythms. Green and blue cells depict SCN neurons and astrocytes, respectively.

Furthermore, the tissue-level organization confers a unique resilience to circadian alterations in the SCN, which does not occur at the single cell level, nor in any other organ (see Figure 1). As such, intercellular communications in the SCN are key for the robustness of the central circadian pacemaker. Indeed, cell–cell interactions in the SCN compensate for cell-autonomous clock phenotypes, due to the loss of function of circadian clock genes such as Cry1 or Per1. For example, loose and low-amplitude circadian rhythms in clock gene expression observed in dispersed cell cultures (either fibroblasts or SCN neurons) from Cry1−/− mutant mice appear to be fully stabilized and synchronized in SCN slices [14]. Even more surprisingly, quasi-circadian oscillations can also emerge from the SCN network itself, when the cell-autonomous clockwork is abolished by the genetic invalidation of the critical clock gene Bmal1 [58]. Thus, the cell-autonomous circadian oscillators assemble within the specific network architecture of the SCN that sustains and amplifies rhythmicity.

Importantly, a primary cause for jetlag and other troubles associated with circadian misalignment resides in this functional cell–cell organization of the SCN, as mice with altered SCN cell synchrony exhibit faster entrainment than control animals to a new lighting schedule. The signaling pathways associated with VIP or AVP, two major neuropeptides produced and secreted by SCN neurons, appear to mediate circadian inertia through SCN cell synchrony, as revealed by the phenotype of mice with null mutations of genes encoding VIP or its receptor VIPR2 [59,60], or the AVP receptors V1a and V1b [61]. While the phenotype of Vip−/− and Vipr2−/− mice may be confounded with a masking effect of light, rather than true circadian entrainment because of their lack of a functional circadian clock, V1a−/− V1b−/− mice unambiguously display an active circadian clock and adjust immediately to abrupt phase advances and delays. In addition, the same resistance to jetlag occurred in response to the pharmacological blockade of AVP signaling in the SCN [61]. Hence, jetlag originates, at least partly, from the SCN, and depends on its cell network organization.

Further support for this specific property of the SCN cell network comes from its resilience to circadian entrainment by temperature. While physiological increases in temperature induce the heat-shock pathway and reset circadian rhythms in pharmacologically-disconnected SCN neurons, as in any other cell type or organ [37,62], temperature cycles appear unable to entrain clock gene expression in cultured SCN slices with preserved cell communications [63,64]. More specifically, this unique resistance of the SCN to temperature requires coupling between its dorsal and ventral parts [64], and it is not observed in SCN slices from neonate animals [65]. Altogether, these data indicate that the specific organization of cell communications in the mature SCN network is responsible for the robustness of the circadian pacemaker and its unique resilience to external perturbations.

## 5. Complexity of Circadian Entrainment in The Integrated SCN

The interaction between circadian rhythmicity in the SCN and temperature, however, appears to be more complex. Intriguingly, heat pulses are able to phase shift the circadian rhythm in SCN neuronal firing of action potentials, even when applied to slices from adult animals [66]. At first glance, this observation may seem to be in contradiction with the well-known resilience of the SCN clockwork to temperature, as described above [63,64]. Yet, this apparent divergence [67] may, rather, underscore differences between the various SCN rhythms in their immediate sensitivity to temperature, with circadian clock gene expression being resilient to temperature fluctuations and the global rhythm in electrophysiological firing rate exhibiting phase entrainment in response to heat.

**Figure 2:**
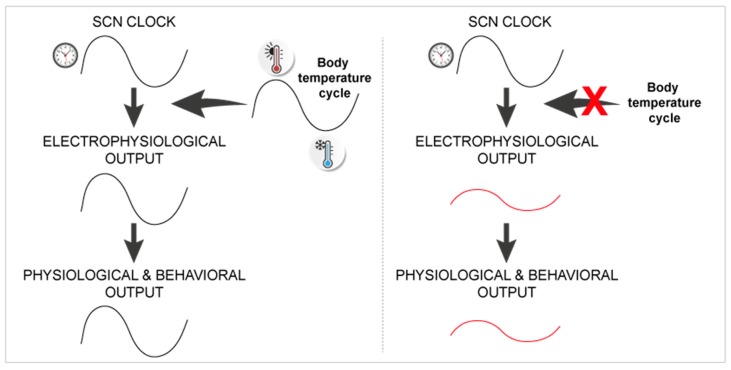
Temperature as a possible capacitor of circadian rhythms. Body temperature cycles may modulate SCN physiology downstream of circadian clock genes, and enable full-amplitude output rhythms in vivo (left panel) that may possibly be reduced in Hsf1−/− mice with an altered heat shock response pathway, but a normal SCN molecular clockwork (right panel).

Recently, we investigated this hypothesis with a mathematical model, assuming a dual regulation of the rhythmic electrical output of the SCN, by the molecular clockwork on one hand, and by the circadian variations in body temperature, on the other hand [68]. We considered the resilience of clock gene expression to temperature [63,64] by applying the effect of temperature downstream of the clock (see Figure 2), as a modulation of the functional link between electrical firing and clock genes [47,51,52]. This very simple relationship permitted us to visualize that temperature cycles may, indeed, entrain the electrophysiological activity rhythm in the SCN without altering the phase of clock gene expression, although we made no assumption regarding the mechanisms actually involved in this differential regulation [68].

The heat shock pathway might be a likely candidate to mediate the effect of temperature on the circadian rhythm in SCN firing of action potentials. Indeed, suppressing the action of temperature in our model of the SCN predicted circadian outputs with smaller amplitudes but a longer free-running period [68], as was observed experimentally in Hsf1−/− mice [69,70]. In line with this hypothesis, the invalidation of the heat shock response also reduces circadian rhythmicity in flies, without altering clock gene expression [71], and a recent study demonstrated that the concerted activity of the circadian clock and heat shock pathways modulates the expression of downstream products in *Drosophila* clock neurons [72]. More generally, the action of body temperature cycles, or any other rhythmic systemic cue, at the SCN level downstream of clock genes may account for the circadian alterations reported under various physio-pathological conditions. For example, the low amplitude in daily body temperature rhythms, as measured in animals eating a cafeteria diet [73] or in lactating female mice [68], is likely responsible for their reduced rhythmic activity of the heat shock pathway in the SCN and, ultimately, for the dampened rhythms in their electrical firing or locomotor activity, despite normal central clock gene expression. This may also account for the reduced circadian output in the ageing SCN [74]. Likewise, it is tempting to speculate that similar mechanisms may also contribute to the chronobiotic effect of melatonin, since melatonin resets the SCN electrical rhythm [75] faster than the expression of most circadian clock genes [76,77].

Furthermore, our hypothesis that temperature cycles modulate circadian SCN output also provides new insight into how jetlag emerges within the SCN. Several studies have previously reported observations that the circadian phase of clock gene expression at the global SCN level may adjust, almost immediately, to a new lighting schedule, while the behavioral rhythm or the circadian rhythm in aggregate SCN electrophysiological activity would show more inertia [78,79,80]. A jetlag simulation in our model accurately recapitulated this dissociation between the SCN clock and output rhythms [68]. This observation supports further the postulate that temperature differentially regulates SCN rhythms, and is in line with the long-standing idea that the circadian pacemaker may reset faster than the locomotor activity rhythm [78,81]. For the sake of simplicity in our model, we considered gene expression and electrophysiological firing as global rhythms in a theoretically homogenous SCN [68]. Future work will address the respective role of separate cell types or of compartments of the SCN in these interactions between external and internal cycles that shape circadian rhythmicity.

**Figure 3:**
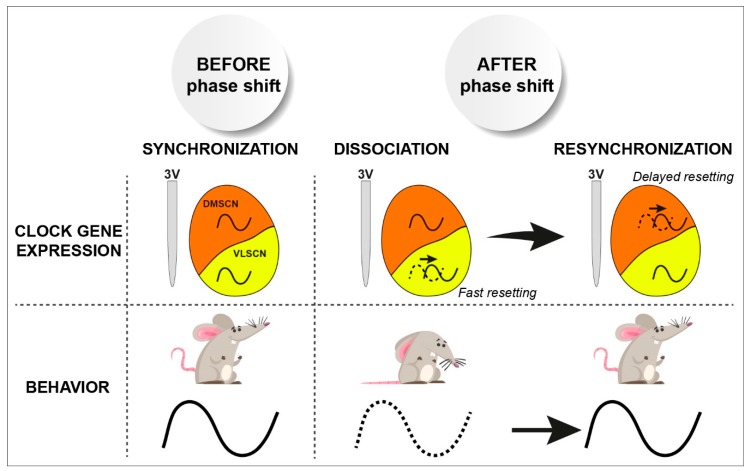
Phase dissociation in the SCN and jetlag. An abrupt light phase shift induces fast resetting of circadian clock gene expression in the ventrolateral part of the SCN (VLSCN) while the dorsomedial part (DMSCN) adjusts progressively to the new lighting schedule, as does locomotor behavior. This transition period before full re-synchronization of the entire SCN corresponds to jetlag (adapted from [83]).

Indeed, spatially resolved investigations revealed that SCN sub-regions reset differentially after an abrupt light phase shift. Clock gene expression entrains almost immediately in the retinorecipient ventral SCN, while the dorsomedial part requires several days to resynchronize (see Figure 3) [82,83,84]. This internal dissociation between SCN regions appears even more complex at the level of electrophysiological activity. Rapidly after phase shift, both the ventral and dorsal parts exhibit a bimodal firing pattern, with an unshifted component and a shifted component that progressively merge into one single shifted peak of activity [85]. Yet these shifted and unshifted components appear solely in the ventral and dorsal SCN, respectively, after application of the gamma-aminobutyric acid (GABA) receptor antagonist bicuculline or a physical cut between both regions [85]. This indicates that the ventral part resets faster than the dorsal part, but that each part tends to impose its rhythm through reciprocal GABAergic communications. Altogether, these data reveal the complexity of circadian phase resetting, and emphasize the SCN origin of jetlag (Figure 3).

## 6. Topological and Functional Complexity of The SCN

The complex and heterogeneous structure of the SCN is characterized by a histological diversity that underlies functional specificity. While all SCN neurons rely on GABAergic neurotransmission, they also synthesize and release a number of peptides involved in cell communications within the SCN and with other brain areas [86,87]. The discrete production of these various neuropeptides defines topological sub-divisions of the SCN, such as the major sub-division into core and shell, as chiefly delineated by the presence of neurons producing VIP and AVP in the ventral and dorsal parts of the SCN, respectively [86,88]. The production of many other neuropeptides, by AVP and VIP neurons or by other SCN neurons, provides a refined view of the cytoarchitectural organization of the SCN [86], and high-dimensional gene expression analysis promises to map the SCN cell diversity at a single-cell resolution [89,90,91].

The discovery of new molecular markers of neuronal diversity in the SCN enables to interrogate the functional relevance of SCN cell types in circadian timekeeping. Importantly, combinatorial genetic experiments revealed that the pacemaker property of the SCN might not rely upon a single neuronal cell type. A recent study provided evidence that SCN neurons expressing the peptide neuromedin S (NMS) may act as possible circadian pacemakers [92]. Indeed, abolishing their molecular clockwork or expressing a long-period circadian mutation specifically in these neurons, respectively, disrupted or lengthened the free-running period of the circadian rhythm in locomotor behavior [92]. NMS-producing neurons encompass a large majority of the AVP and VIP neurons, as well as other neuronal cell types, with the remarkable exclusion of neurons containing the gastrin-releasing peptide. However, neither AVP nor VIP neurons seem capable of imposing their own period onto the body rhythms [92,93]. Thus, the circadian phenotype communicated by NMS neurons to the rest of the organism may either be due to AVP-negative and VIP-negative neurons expressing NMS, or result from a non-specific mass effect; simply because NMS neurons represent a large proportion (40%) of SCN neurons. Two different studies, conducted with other mouse models, support this latter hypothesis. Firstly, the use of chimeric mice randomly expressing an untargeted circadian mutation revealed that the period of their behavioral rhythm directly depends on the proportion of SCN neurons, with a long or short circadian period [94]. Secondly, the specific targeting of circadian mutations to another subset of SCN neurons, which express the dopamine receptor DRD1A, produced mice with an unstable free-running period that revealed the competition between DRD1A-positive and DRD1A-negative neurons in setting the ensemble period [95]. In other words, each set of SCN neurons could alternatively impose its own period. Hence, the pacemaker activity of the SCN emerges from different neuronal populations interacting in a plastic cell network.

In addition, the multi-cellular functioning of the SCN further increases if one considers that the circadian pacemaker may not rely solely upon purely neuronal processes. Like neurons, astrocytes contain the cell-autonomous molecular clockwork [96]. Remarkably, these glial cells regulate daily behaviors by controlling circadian timekeeping in the SCN. Pioneering work suggested that the timely coverage of dendrites by astrocytes may contribute to the daily synaptic plasticity in the SCN [97], and more recent studies showed that the targeted inactivation of the molecular clock in SCN astrocytes lengthened the circadian period, ex vivo and in vivo [98,99]. Moreover, SCN astrocytes exhibited circadian variations in cytosolic calcium concentration in antiphase with neurons and contributed to the nocturnal silencing of neuronal activity by regulating extracellular glutamate levels [100]. This mechanism enables astrocytes to communicate their own tempo to the SCN, and to play the role of circadian pacemaker in absence of other clock cells [101]. Altogether, these data indicate that glial transmission interacts with neuronal signaling to regulate circadian activity in the SCN. Whether several astrocyte sub-types exist and regulate different neuronal cell types to generate specific neuron-glia networks in the SCN remains unknown.

Importantly, because voluntary locomotor rhythmicity is commonly the single measured circadian output, one may tend to generalize behavioral observations and assume that one specific SCN cell type must regulate all circadian rhythms. Yet, although the SCN coordinate all bodily rhythms, they may contain distinct circadian pacemakers dedicated to specific functions. As soon as the study of human chronobiology emerged, it was observed that physiological and locomotor rhythms free-ran with different periods in the same subject [102]. In rodents, the exposition to short (22 h) light/dark cycles [103], or the selective lesion of SCN fibers projecting to the subparaventricular zone (SPZ) of the hypothalamus [104], or the suppression of GABA signaling in the SPZ led to a dissociation between internal body temperature and behavioral rhythms. Therefore, sets of cells projecting towards a given brain area and involved in pacing a same biological rhythm may define functional units of the SCN. As SCN cells with different neurochemical phenotypes or locations happen to project efferent fibers to a same target brain area [87,105], these functional units could significantly differ from the currently proposed sub-divisions.

An interesting example of functional topology in the SCN resides in the regulation of growth hormone (GH) secretion. GH production by somatotroph cells in the anterior pituitary gland, and its release into the blood circulation, are under the dual control by somatostatin (SMS) and GH-releasing hormone (GHRH), from hypophysiotrophic endocrine neurons in the hypothalamus. The alternate release in the median eminence of these two inhibiting and stimulating neurohormones, respectively, results in a characteristic and highly pulsatile ultradian pattern in blood GH concentration, which appears disrupted in circadian clock mutant mice [106]. It is worth noting that the SCN regulates both SMS and GHRH productions. A pool of SCN neurons mediates photic information to SMS-producing neurons in the periventricular nucleus of the hypothalamus [107]. This connection is responsible for the daily inhibition of GH secretion at the onset of light, and for the ensuing synchronization of ultradian GH pulses with the light-dark cycle [107,108]. In addition, a subset of VIP-containing SCN neurons, which specifically express the neuropeptide Y/pancreatic polypeptide receptor Npyr6, regulate GHRH production and contribute to body growth and metabolism [109]. Importantly, the SCN balance upon SMS and GHRH neurons may be impaired during circadian perturbations, as suggested by the abolition of pulsatile GH secretion in a rat model of night-shift work [110]. Although further experiments will be necessary to decipher the functional links between both pathways, the concerted action of different SCN neurons results, here, in temporally-organized functional activity at the organism level.

## 7. Exploring The SCN Cell Network Organization

How multiple cell-autonomous oscillators interact in a coherent circadian pacemaker remains largely unknown. Stable, real-time imaging of clock gene expression, membrane voltage, or cytosolic calcium concentration within slices, has already revealed important features of the spatio–temporal organization of circadian rhythms throughout the SCN (as reviewed in [57]). Remarkably, a complex interaction between synaptic communication, mostly involving GABA [85,111,112], and paracrine regulation by diffusive factors [113] results in local synchronization and long-distance network coordination of cell rhythms, which, thus, gives rises to travelling waves from one side of the SCN to another [114,115,116,117]. The robustness of the circadian pacemaker undoubtedly stems from this tissue-level organization. Finding proper tools to cure jetlag thus requires deciphering not only the precise synaptic wiring between neurons, but also the mechanisms underlying the ordered, remote volume transmission involving multiple SCN cell types. As the expression of transmitters and their receptors marks the largest difference between SCN sub-divisions [90], spatially resolved genome-wide transcriptional profiling [118] may now make it possible to map all the cells able to communicate with one another. Such molecular maps would be ideal tools to direct the functional assessment of each emitter/receptor cell pair.

Importantly, the presence of highly connected “hub” cells unveils privileged nodes of the SCN network. The application of tetrodotoxin desynchronizes unitary cell rhythms within SCN slices [15], and the monitoring of their resynchronization after washing provided an elegant way to infer the topology of the network [119,120]. Remarkably, this analysis revealed different kinetics of stabilization across the slice [120], and the characteristics of a small-world organization, with densely connected cells in the core SCN surrounded by sparsely connected shell regions [119]. Further characterization of these hub cells would enable to assess their particular role in the SCN. For example, the identification of specific molecular markers would, then, make it possible to probe whether these cells constitute a separate cell type, and whether they are critical for the coordination of clock cells and the robustness of the SCN pacemaker. However, although other work supports the small-world topology [121], this organization has been observed in organotypic slice cultures, obtained from immature SCN. It still remains to ensure that it also holds true in the mature hypothalamus. Moreover, SCN slices and explants represent genuine paradigms to probe cell networks ex vivo, but these approaches may also damage important connections that would be important for capturing the complete picture of what eventually happens in the intact SCN, such as in vivo.

Conversely, monitoring SCN rhythms in vivo, at a cellular resolution, has long proved challenging. Extracellular recording of neuronal firing [122,123,124] and photometric measurements of bioluminescent or fluorescent reporters [125,126] provide a global view of SCN rhythms at the cell population level, in both anesthetized and freely-moving animals. Although these approaches hardly permit to infer single cell activity, the combination of in vivo photometry with specific targeting of genetically-encoded indicators enables to probe, in real time, the activity of different cell types in the SCN of adult animals. A recent study demonstrated that this technique permits stable recordings of clock gene expression in the mouse SCN over several months, and unambiguously confirmed that the molecular clock in VIP-producing neurons resets at least twice faster than the running-wheel behavior after a light phase shift [127].

Similarly, in vivo monitoring of calcium activity with the fluorescent probe GCamp6s, specifically targeted to VIP-producing neurons, enabled an assessment of the responsiveness to light of the retino-recipient cells in the SCN [128]. It is worth noting that not only did integrated calcium levels exhibit slow variations at the circadian timescale, but also had higher average concentrations measured during the light phase, as one would have expected [129]; but fast events of increased fluorescence for a few seconds also emerged from the global signal during the day [128]. These rapid variations at the cell-population level were very likely the fingerprint of the coordinated calcium activity among a large number of VIP neurons in the SCN, which suggests the dynamic coupling and uncoupling of SCN cells during the course of the day/night cycle. Moreover, the persistence of such signals under constant darkness conditions indicates that the plasticity of this SCN cell network is under the control of circadian clocks.

Strikingly, the investigation of the SCN network dynamics at a cell resolution in vivo has now become possible, thanks to the extreme miniaturization of fluorescence microscopy devices. Recently, a fluorescence microscope, weighing not more than 2 g, has been developed [130,131]. Once cemented onto the skull of small rodents, such as mice, this mini-scope permits to monitor cell activity in superficial brain areas, and gradient-index (GRIN) lenses can establish an optical path toward deeper brain regions, such as the hypothalamus [132,133,134]. Implementing these tools to record SCN activity will offer the unique combination of cell-resolution imaging in slices with the preserved integrity of mature cell networks in vivo. This approach recently permitted an unveiling of how cell modules underlie a social behavior [135]. It will, undoubtedly, soon help to reveal the secrets of the functional organization of SCN cell networks and their plasticity. More specifically, in vivo calcium imaging will permit to simultaneously interrogate fast cell events, typical of synaptic and paracrine signaling within the SCN network, and the slow variations associated with circadian regulation. This novel knowledge about how the SCN integrates these short and long timescales, respectively, under normal and perturbed conditions, will be key for a better understanding of jetlag and other circadian disorders.

## 8. Conclusions

More than three centuries after the French astronomer Dortous de Mairan reported the first evidence of an endogenous circadian rhythm, chronobiologists have disentangled the molecular cog-wheels of the biological clock. This great achievement, however prodigious, is only a first step toward a full comprehension of the mechanisms of circadian timekeeping. It is now time to understand how cell-autonomous oscillators team up into a robust circadian pacemaker [56,57]. What are the functional modules that confer the features of resilience and robustness to an ensemble of clock cells that individually lack these properties [64]? Circadian drosophilists opened the path and investigated the functional topology of cell networks which organize circadian timekeeping and photic entrainment in whole-brain preparations or living flies [136,137,138,139]. The translation of these approaches to mouse models will reveal the circuit architecture of the mammalian SCN and its contribution to jetlag.

## Abbreviations

SCN	Suprachiasmatic nuclei
TTFL	Transcription-translation feedback loops
CCG	Clock-controlled gene
VIP	Vasoactive intestinal polypeptide
AVP	Arginine Vasopressin
GABA	Gamma-aminobutyric acid
SPZ	Subparaventricular zone
GH	Growth hormone
SMS	Somatostatin
GHRH	GH-releasing hormone
